# Unexplained Hyperthyrotropinemia: A Biochemical and Clinical Challenge

**DOI:** 10.3390/jcm12082934

**Published:** 2023-04-18

**Authors:** Laura Croce, Spyridon Chytiris, Francesca Coperchini, Giovanni Ferraro, Linda Minelli, Antonella Navarra, Flavia Magri, Luca Chiovato, Pierpaolo Trimboli, Mario Rotondi

**Affiliations:** 1 Department of Internal Medicine and Therapeutics, University of Pavia, 27100 Pavia, Italy; 2 Unit of Endocrinology, Laboratory for Endocrine Disruptors, Istituti Clinici Scientifici Maugeri IRCCS, 27100 Pavia, Italy; 3 Laboratory Service, Istituti Clinici Scientifici Maugeri IRCCS, 27100 Pavia, Italy; 4 Unit of Internal Medicine, Medical-Oncologic Department, ASST Lodi, 26900 Lodi, Italy; 5 Clinic for Endocrinology and Diabetology, Lugano Regional Hospital, Ente Ospedaliero Cantonale, 6900 Lugano, Switzerland; 6 Faculty of Biomedical Sciences, Università della Svizzera Italiana (USI), 6900 Lugano, Switzerland

**Keywords:** unexplained hyperthyrotropinemia, macro-TSH, subclinical hypothyroidism, laboratory interference

## Abstract

Background: A raised serum TSH in the absence of a clear etiology, or “unexplained hyperthyrotropinemia” (UH), can be challenging for clinicians. The aim of the present study was to evaluate potential strategies aimed at a clinical and biochemical characterization of UH patients. Methods: We compared 36 patients with UH with a control group of 14 patients with chronic autoimmune thyroiditis (CAT) and subclinical hypothyroidism. The two groups were compared in terms of the following: (i) the rate of normalization of TSH after repeating with another assay; (ii) the rate of normalization of TSH over time with the same assay; (iii) the reduction in TSH after precipitation with polyethilenglycole (PEG); and (iv) free thyroxine (FT4) levels. Results: Similar TSH levels were observed in UH [5.65 (5.21–6.37)] and CAT [5.62 (5.17–8.50)] (*p* = 0.489). TSH measurement with another assay method showed a normal TSH value in 41.9% of UH vs. 46.1% of CAT patients (*p* = 0.797). After repeating the TSH measurement in time with the same assay method, an increased TSH value was confirmed in all cases, in both groups (0% in the UH group vs. 0% in the CAT group, *p* = 1.000). TSH recovery after PEG precipitation was similar in the two groups (% precipitable post-PEG: 68.75 ± 3.14 in UH vs. 68.67 ± 7.18 in CAT, *p* = 0.960). FT4 levels were similar in the two groups (FT4 1.02 ± 0.20 ng/dl in UH vs. 1.00 ± 0.20 ng/dl in CAT, *p* = 0.789). Conclusions: The results do not support the concept that laboratory interferences are more frequent in UH patients, suggesting that patients with UH should be managed in the same way as patients with CAT until proven otherwise.

## 1. Introduction

TSH measurement is the first-line screening test for thyroid dysfunction. An elevated serum level of TSH is, in most cases, indicative of hypothyroidism, which is further defined as subclinical or overt according to concomitant normal or reduced free thyroxine levels [1]. Chronic autoimmune thyroiditis (CAT) represents the most common etiology sustaining hypothyroidism. Over recent decades, several other clinical conditions have been characterized, including the serum-negative chronic autoimmune condition, which, according to most recent case series, is observed in nearly 20% of hypothyroid patients [2]. More recently, the concept that a raised serum TSH might not always be suggestive for hypothyroidism was proposed, as in the case of morbid obesity [3]. In addition to morbid obesity, there are several other situations in which a raised serum TSH might be difficult to be ascribed to a precise cause, sustaining the concept that a few patients may actually have a rather “unexplained hyperthyrotropinemia” (UH). These include the rare possibility of laboratory interferences, which should be suspected in particular when TSH measurement results are highly discrepant between different TSH assays and/or when nonlinearity is observed after serial dilution [4]. Among the possible causes of assay interference, heterophile antibodies (HAbs) [5,6], anti-rhuthenium or anti-biotin antibodies [7,8,9,10] and TSH variants [11] account for most of these rare situations. More recently, the presence of macro-thyrotropin (macro-TSH) was identified as a possible cause of falsely elevated TSH levels [12]. Macro-TSH is caused by multiple monomeric TSH molecules complexed with anti-TSH antibodies, mostly immunoglobulin G [13,14]. Biochemically, this hyperthyrotropinemia mimics subclinical hypothyroidism, potentially leading to misdiagnosis and/or to inappropriate LT4 therapy. Although the presence of macro-hormones could easily be ruled out by gel filtration chromatography (GFC), this is not routinely available in most laboratories. A surrogate measure could be represented by the use of polyethylene glycol (PEG)-mediated precipitation, which can much more easily be performed in most laboratories. Indeed, post-PEG recovery level of monomeric prolactin and the establishment of laboratory-specific post-PEG monomeric prolactin reference intervals [15] has been shown to be effective. Given the similarity between macro-PRL and macro-TSH, dilution studies and the measurement of TSH after the addition of PEG were suggested as potentially helpful tools to identify potential assay interference. The sharp increase in the number of prescriptions of TSH measurements as a result of screening campaigns (e.g., pregnancy and obesity, among the more common ones) has led to a parallel increase of the biochemical finding of UH.

Thus, it could be hypothesized that macro-TSH could be more prevalent than hitherto reported when a selected population (i.e., patients with UH) is taken into account. Theoretically, the diagnostic challenge might be easily addressed by using GFC, but this remains unavailable in most laboratories, and clinicians have to rely on alternative tools.

The aim of the present study was to evaluate the implications of potential strategies aimed at a more precise characterization of UH patients.

## 2. Materials and Methods

The study group included consecutive patients with “unexplained hyperthyrotropinemia” (UH) attending the endocrinology clinic of ICS Maugeri of Pavia from July 2020 to August 2022.

UH was defined as the presence of a raised serum level of TSH in the absence of the following: (i) positive tests for thyroid autoantibodies; (ii) hypoechoic pattern of the thyroid at ultrasound; (iii) concurrent clinical conditions and/or drugs potentially related to isolated hyperthyrotropinemia; and (iv) BMI > 30 kg/m^2^. 

The control group was constituted by sex- and age-matched patients with raised serum TSH in whom a complete thyroid work-up (thyroid auto Ab and ultrasound) led to an established and firm diagnosis of CAT.

Common inclusion criteria included subclinical hypothyroidism (as defined by a raised serum TSH with normal FT4 levels).

Common exclusion criteria were as follows: (i) post-thyroidectomy and/or radioiodine hypothyroidism; (ii) therapy with thyroid interfering drugs (i.e., amiodarone, lithium); (iii) acute illness at the moment of sampling; (iv) pregnancy or post-partum thyroiditis; (v) morbid obesity; and (vi) cystic fibrosis.

For all patients, a blood sample was obtained to determine the value of TSH and TSH after PEG-mediated precipitation at our central laboratory.

The TSH assay used was the Alinity I system (Abbott Laboratories, Lake Forest, IL, USA) which is an automated analyzer that utilizes the chemiluminescent microparticle immunoassay (CMIA) principle, by using anti-analyte coated paramagnetic microparticles and anti-analyte acridinium-labeled conjugates. The reaction is measured as relative light units, which have a direct relationship with the amount of analyte in the sample. Serum samples (50 mL) were then treated with 50 µL of 25% PEG (final concentration of PEG: 12.5%) to precipitate γ-globulin fractions, and with 50 µL of water for total TSH. TSH in the supernatant of the PEG-treated sera and water-treated sera was designated as free and total TSH, respectively. The PEG-precipitable TSH (%), which may be increased in sera containing macro-TSH, was calculated as follows: (total TSH—free TSH)/total TSHx100). Normal ranges for TSH were 0.35–4.95 µUI/mL

The alternative TSH assay used was the TSH3-Ultra assay on the ADVIA Centaur XP analyzer (Siemens Healthineers USA, Malvern, PA, USA) which is an automated analyzer that utilizes an anti-TSH capture monoclonal antibody marked with FITC and a bovine serum albumin (BSA) conjugate monoclonal antibody for the chemiluminescent assay. The reaction is measured as relative light units, which have a direct relationship with the amount of analyte in the sample. Normal ranges for TSH were 0.55–4.78 µUI/mL.

### Statistical Analysis

Statistical analysis was performed using SPSS software version 20 (SPSS, Inc., Evanston, IL, USA). Between-group comparisons were performed using Student’s t-test for unpaired data and the Mann–Whitney U test, according to a normal or a nonparametric distribution of the variable tested. Paired samples comparisons were performed using a paired samples t-test and the Wilcoxon rank test, according to a normal or a nonparametric distribution of the variable tested. Correlation between two variables was ascertained using Pearson and Spearman correlation tests, as appropriate. Frequencies among groups were compared using a χ^2^ test with Fisher’s correction, when appropriate. A *p* value < 0.05 was considered statistically significant. Variables were reported as mean ± standard deviation (SD) in the case of normally distributed variables and as median (interquartile range) in the case of non-parametric distribution.

## 3. Results

The sonographic and biochemical characteristics of the two groups are described in Table 1. The study group (UH) included 36 patients with no sonographic or biochemical features of chronic autoimmune thyroiditis (study group, UH). The control group included 14 patients with an established diagnosis of chronic autoimmune thyroiditis (control group, CAT). A total of 8/36 patients from the UH group and 6/14 patients from the CAT group had been previously treated with thyroid hormone replacement therapy; all of them were asked to stop the replacement therapy at least 3 months before the time when the blood sample for PEG treatment was collected.

The two groups were similar in terms of age and sex. TSH levels and FT4 levels were similar between the two groups.

To verify the TSH values, in 44 patients (31 in the UH group and 13 in the CAT group) the TSH measurement was repeated using a different assay method. Among these patients, in 13/31 patients (UH) and 6/13 patients (CAT) a normal TSH value was obtained after repeating the TSH measurement in another laboratory (moving from a median of 5.50 (5.00–6.37) to a median of 2.80 (2.55–3.51)). For the remaining patients (18/31 in the UH group and 7/13 in the CAT group), an increased level of TSH was confirmed even after repeating the measurement in another laboratory by a different assay method (ADVIA Centaur^®^ XPT Immunoassay System by Siemens) (a median of 5.64 (5.24–7.80) at first measurement vs. a median of 5.45 (4.32–6.59) at second measurement).

Among the patients in which an increased TSH level was confirmed after repeating the assay with another assay kit, 21 patients (16/36 in the UH group and 5/14 in the CAT group) underwent a repeated TSH measurement with the same assay kit after a minimum period of 2 months. For all these patients, an increased TSH level was confirmed after repeating the measurement (moving from a median of 6.20 (5.50–7.81) to a median of 5.63 (4.96–6.59)).

**Figure 1:**
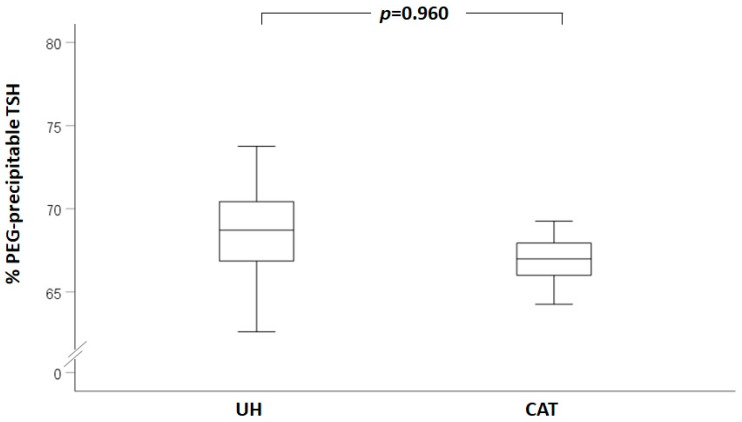
Box plot representing the percentage of PEG-precipitable TSH in the unexplained hyperthyrotropinemia (UH) and chronic autoimmune thyroiditis (CAT) groups.

When the PEG-mediated precipitation was performed, TSH recovery was similar between the two groups (% precipitable after PEG: 68.75 ± 3.14 in the UH group vs. 68.67 ± 7.18 in the CAT group, *p* = 0.960, as illustrated in Figure 1). We also performed a comparison of results of PEG precipitation between the 19 patients with a different result between the two methods and the other patients. This comparison also showed no significant difference in post-PEG reduction of TSH between the two groups (69.39 ± 5.72% in those with discrepant results vs. 68.32 ± 3.71% in those with no discrepant results, *p* = 0.428). We also compared the UH and CAT groups including only patients with discrepant results, but again we saw no significant differences between the two study groups (69.02 ± 3.29 in the UH group vs. 70.17 ± 9.53 in the CAT group, *p* = 0.696).

Among the enrolled patients, only two of the CAT group and one of the UH group PEG-precipitable ratios were > 75%, thus mildly suspicious for macro-TSH (patients’ characteristics are shown in Table 2). All three of these patients underwent a repeated TSH measurement in another laboratory with the ADVIA Centaur^®^ XPT Immunoassay System by Siemens. Repeating the TSH measurement resulted in a normal value in two patients (one in the UH group and one in the CAT group) and in an elevated value in the remaining patient (in the CAT group).

## 4. Discussion

The present study was designed with the aim of evaluating possible diagnostic strategies, simple and readily available for clinicians, to face the diagnostic challenge of a patient presenting with a raised serum level of TSH in the absence of any detectable thyroid disease and/or underlining clinical condition potentially justifying the biochemical finding. The above-mentioned condition remains challenging in that the clinical question to be addressed is whether that patient is affected by subclinical hypothyroidism and whether appropriate LT4 replacement therapy should be initiated. Potential laboratory interferences in the measurements of TSH are often advocated, but these might not always be easily ascertained. Recently, the possibility that a “falsely” elevated TSH level could result from the presence of macro-TSH has been proposed. However, the problem remains, since, as reported by several authors addressing the issue [16,17], there are at present no reliable assay methods for ruling out such a possibility. Clearly, the exception would be provided by GFC, but the lack of routine availability of this method in most clinical laboratories has left the problem unsolved in the vast majority of cases. Thus, our working hypothesis was to surrogate measures readily available in a “real life” clinical setting that could be helpful in the absence of GFC. In this view, the possibility of comparing the findings obtained in a group of patients with UH as opposed to CAT patients provided helpful clinical information.

From the clinical standpoint, the herein reported results prompt discussion of four points.

First, repeating a TSH measurement in a different laboratory using a different assay method, which is often advocated as a simple measure to identify a potential laboratory interference [4], allowed the obtention of a normal TSH value in most subjects, without significant differences between UH and CAT. Obtaining different results on different measurement platforms, especially in cases of only mild elevations of TSH, is a rather frequent clinical problem that can be cumbersome for the clinician. Nevertheless, the fact that this phenomenon occurred in a similar percentage of patients in the UH and CAT groups suggests that UH would not be enriched in patients with a possible assay interference. Moreover, the stratification of results according to the presence or absence of discrepancies between laboratories did not reveal any differences in the values of post-PEG precipitation rates in the two groups.

Second, repeating TSH measurement over time with the same assay method to rule out fluctuations in the TSH circulating levels, according to the most recent clinical guidelines on subclinical hypothyroidism, did not unveil different results between UH and CAT [18,19], and was not helpful in our series of patients.

Third, TSH measurement after PEG treatment showed overlapping percentages of PEG-precipitable TSH between the CAT and UH groups (68.75 ± 3.14 in the UH group vs. 68.70 ± 7.18 in the CAT group), warranting a specific discussion. The above values are similar to those reported in normal controls without macro-TSH by Hattori et al., when using the same TSH assay used in the present study [16]. Indeed, the results of PEG precipitation on TSH values vary significantly according to the measurement assay employed, similar to the results observed in the context of insulin measurement in patients with Hirata syndrome [20], and thus should be interpreted with caution, and always by using laboratory-specific ranges. Unlike the measurement of prolactin, for which a PEG-precipitable PRL > 60% (or a recovery < 40%) is usually used as a threshold reliably indicating the presence of macro-prolactinemia [21], to date, there is not a validated cut-off of PEG-precipitable TSH to define the presence of macro-TSH. Hattori et al. proposed that the presence of macro-TSH should be strongly suspected when PEG-precipitable TSH ratios were > 90% or when necessary, even if the ratios were between 75 and 90% [14]. The same group of researchers recently reported that, among 138 serum samples of neonates with a PEG precipitation ratio of TSH > 68.9%, macro-TSH was found in 0.43% of cases [22]. No definite indications exist regarding the need for screening for macro-TSH, a condition generally considered to be rare [13,14,16,17,23]. Some authors suggest screening only patients with a TSH value above 10µU/mL and normal FT4 levels [16], although cases with lower TSH values have been reported [12].

Thus, an attempt to separately evaluate outlier patients for PEG results (PEG-precipitable TSH > 75%) was performed. A percentage of precipitation above 75% was observed in only one patient in the UH group and in two patients in the CAT group. It should be noted that in two of these cases (one in the UH group and one in the CAT group), a normal TSH level was obtained using an alternate platform, even before PEG precipitation, suggesting that the possible assay interference was solved simply by changing the assay method. It should also be noted that, unlike most of the patients with macro-TSH described in the previously published case reports and case series, none of these three patients had a markedly increased TSH.

Fourth, the last attempt to highlight differences between UH and CAT patients was performed by comparison of FT4 levels. Indeed, given the hypothesis that CAT patients would be truly hypothyroid while bioassay interference would characterize patients with UH, one would expect lower and higher levels of FT4 in the two groups, respectively. However, similar FT4 levels characterized UH and CAT patients, indirectly indicating that the UH group did not include a higher number of “false hypothyroid” patients than the CAT group.

In synthesis, none of the above reported strategies was able to discriminate between UH and CAT patients, who reported overlapping results using any of the above procedures. Even if in a relevant percentage of patients a normal TSH level was obtained simply by repeating the exam on another platform, the lack of differences between the UH and CAT groups suggests that assay-related issues are not a major determinant of high TSH levels in the UH group. Although it should be made clear that at present GFC remains the gold standard to diagnose macro-TSH, some conclusions stemming from our results should be highlighted. Indeed, the above results do not support the hypothesis that macro-TSH may be a major cause sustaining UH.

The overlapping values of FT4 represent a strong argument against the hypothesis that, while CAT patients would be “truly” hypothyroid, UH would be euthyroid patients with a falsely elevated TSH. This finding appears to have non-negligible clinical repercussions, in that we have probably overestimated the possibility of laboratory interference accounting for elevated TSH levels in patients with no definite cause sustaining thyroid deficiency. Among the well-known causes of TSH elevation, medication (i.e., lithium or amiodarone), obesity, post-partum thyroiditis, non-thyroidal illness recovery and cystic fibrosis should be taken into account [1,24]. In our study group, none of the patients presented the conditions mentioned above. Thus, other causes could account for the UH in our study group. A raised serum TSH, in the absence of established etiologies, is generally referred to as idiopathic subclinical hypothyroidism [25], which may encompass a wide spectrum of rare conditions including the following: (i) mutations in genes encoding proteins involved in TSH pathway; (ii) TSH-receptor anomalies; and (iii) alterations in genes encoding proteins involved in the signaling pathway downstream of the receptor, which are thought to be responsible for some cases of idiopathic subclinical hypothyroidism, especially in those cases arising in familial settings [11,26].

Some limitations of the present data should be discussed. First, these data derive from clinical practice, where patients were managed according to their clinical context. Second, no data about free T3 were available to further explain the TSH values. Third, both negative ultrasound and anti-thyroid antibodies might not completely rule out the presence of chronic autoimmune thyroiditis, even if this is a rare scenario.

## 5. Conclusions

In conclusion, the results of the present study, especially in view of the overlapping results in terms of the PEG precipitation of TSH and FT4 between the UH and CAT patients, suggest that macro-TSH is, as previously reported, a very rare clinical condition [12,13,16,27].

Considering the low prevalence of macro-TSH and the diagnostic difficulties in determining it, in the face of blood tests indicative of subclinical hypothyroidism without clues of autoimmune thyroid disease, it remains necessary to search for further causes of hypothyroidism. At present, it remains the case that patients with unexplained hyperthyrotropinemia should be managed in the same way as patients with chronic autoimmune thyroiditis, until proven otherwise.

## Figures and Tables

**Table 1:** Clinical and biochemical characteristics of unexplained hyperthyrotropinemia (UH) and chronic autoimmune thyroiditis (CAT) groups. TSH: thyreotropic hormone. FT4: free thyroxine. LT4: levothyroxine.

	Study Group (UH)	Control Group (CAT)	*p* Value
N	36	14	
Sex (F/M; % of females)	28/8 (77.8%)	9/5 (64.3%)	0.329
Age (years, mean ± SD)	45.4 ± 19.8	46.1 ± 16.9	0.913
TSH at first evaluation (µU/mL, median (interquartile range))	5.65 (5.21–6.37)	5.62 (5.17–8.50)	0.489
FT4 at first evaluation (ng/dl, mean ± SD)	1.02 ± 0.20	1.00 ± 0.20	0.789
Patients previously treated with LT4 (N, %)	8/36 (22.2%)	6/14 (42.9%)	0.145

**Table 2:** Clinical and biochemical characteristics of patients with a PEG-precipitable TSH ratio > 75%. TSH: thyreotropic hormone. FT4: free thyroxine. PEG: polyethilenglycole. BMI: body mass index. AbTPO: anti-thyreoperoxidase antibody. AbTg: anti-thyreoglobulin antibody.

	Patient 1	Patient 2	Patient 3
Group	CAT	CAT	UH
Sex	Male	Female	Female
Age (years)	68	21	74
BMI (kg/m^2^)	24.2	32.4	28.9
TSH—Alinity I system, Abbott (uUI/mL)	5.3	5.1	5.5
TSH—ADVIA Centaur^®^ XPT system, Siemens (uUI/mL)	3.80	5.50	3.50
FT4 (ng/dL)	1.14	0.73	0.90
TSH % precipitable post-PEG	89.28	79.05	77.73
AbTPO	Positive	Positive	Negative
AbTG	Positive	Positive	Negative
Sonographic thyroid features	Markedly hypoechoic and inhomogeneous structure; Normal vascularization.	Markedly hypoechoic and inhomogeneous structure; Slightly increased vascularization.	Normoechoic and homogeneous structure; Normal vascularization.

## Data Availability

The data presented in this study are available on request from the corresponding author. The data are not publicly available due to privacy of the included subjects.
